# Subclinical macro and microvascular disease is differently associated with depressive symptoms in men and women: Findings from the SABRE population-based study

**DOI:** 10.1016/j.atherosclerosis.2020.09.005

**Published:** 2020-11

**Authors:** Jingyi Wang, Therese Tillin, Alun D. Hughes, Marcus Richards, Naveed Sattar, Chloe Park, Nish Chaturvedi

**Affiliations:** a MRC Unit for Lifelong Health and Ageing, Institute of Cardiovascular Science, University College London, London, United Kingdom; b Department of Social Medicine, School of Public Health, Fudan University, Shanghai, China; c Institute of Cardiovascular and Medical Sciences, University of Glasgow, Glasgow, United Kingdom

**Keywords:** Vascular diseases, Depression, Risk factors, Sex differences, Epidemiology

## Abstract

**Background and aims:**

Mechanisms underlying the association between cardiovascular disease (CVD) and depression are unknown, and sex differences understudied. We investigated associations between a comprehensive set of measures of macro and microvascular disease and depressive symptoms in older men and women.

**Methods:**

We performed cross-sectional analyses of the SABRE (Southall And Brent REvisited) population-based study. Participants (1396) attended clinic between 2008 and 2011 for assessment of subclinical macrovascular (carotid ultrasound, echocardiography, cerebral magnetic resonance imaging) and microvascular (retinopathy, nephropathy) disease, and depression.

**Results:**

Mean age of 1396 participants was 69.5 years, and 76.2% were male. The median (interquartile range) of depression score was 1 [0, 2] for men and 1 [0, 3] for women. All measures of subclinical macro and microvascular disease were adversely associated with depressive symptoms, even when known CVD was excluded. Physical activity partly explained some of these relationships. The association between left atrial dimension index (LADI), a measure of chronic elevated left ventricular filling pressure, and depressive symptoms was stronger in women (regression coefficient 0.23 [95% CI 0.11, 0.35]) than men (0.07 [-0.01, 0.15]), *p* for interaction 0.06, on multivariable adjustment.

**Conclusions:**

Subclinical macro and microvascular disease is associated with depressive symptoms, even in the absence of established CVD. These were in part accounted for by physical activity. We observed stronger association between LADI and depressive symptoms in women than in men. The beneficial role of physical activity in abrogating the association between subclinical CVD and depression warrants further investigation.

## Highlights

• Subclinical macro and microvascular disease is associated with depressive symptoms.
• The associations appeared to be present before cardiovascular symptoms develop.
• Physical activity partly explained some of these relationships.
• The associations may be different in men and women.

## Graphical abstract

**Figure:**
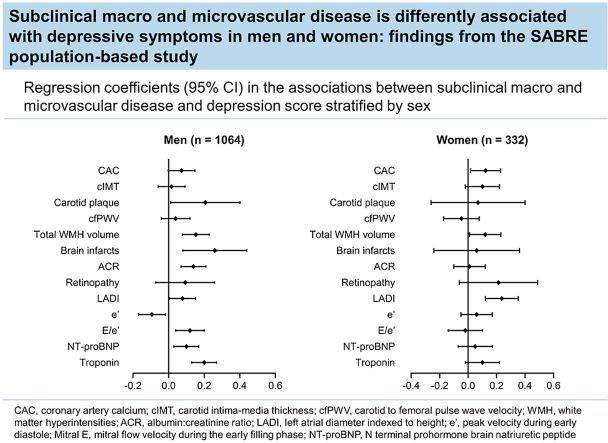

## 1 Introduction

There is an established and possibly bi-directional relationship between cardiovascular disease (CVD) and depression. A fifth of outpatients with coronary heart disease (CHD) report major depression [1], and mild to severe depression is present in about half of all people with heart failure [2]. In contrast, depression is also associated with an increased risk of incident CHD [3,4], and in those with established disease, predicts both morbidity and mortality [5,6].

The underlying mechanisms linking CVD and depression are unclear. Exploration of associations between subclinical and location specific atherosclerosis, and between macrovascular and microvascular disease with depression may provide mechanistic insights. However, findings of previous studies of subclinical atherosclerosis are inconsistent. Coronary artery calcium (CAC), carotid intima media thickness (cIMT) and pulse wave velocity (PWV) have been related to depression or depressive symptoms in some studies [[7], [8], [9]] but not others [[10], [11], [12]]. Cross-sectional and longitudinal associations between white matter hyperintensities and late-life depression have been reported in a meta-analysis [13]. However, data on relationships between other forms of microvascular disease and depression are scarce. Most of these studies focused only on a single component of CVD, and, in older age, some did not exclude individuals with established CVD. Men and women differ in both their cardiovascular risk profiles/events and depressive symptoms. Subclinical atherosclerosis appears to be more frequent among men than women [14,15] as well as incident major adverse cardiovascular events particularly CHD [16], whereas women have poorer left ventricular diastolic function [17,18] with heart failure as the dominant type of cardiovascular event in older age [16]. Depression is known to be more prevalent in women than in men [19]. However, few studies have stratified their analyses by sex, and findings so far are inconsistent: higher subclinical atherosclerosis was associated with more severe depressive symptoms among men but not women in three studies [7,20,21] and in another study a negative association between CAC and depression was found in women and a U-shaped association in men [22]. These studies only used a single measure of CVD and thus are unlikely to fully characterise sex differences in comorbid CVD and depression.

We conducted a cross-sectional analysis to investigate the association between a comprehensive set of measures of macro and microvascular disease and depressive symptoms in a community-based population of older men and women with and without diagnosed cardiovascular disease.

## 2 Materials and methods

### 2.1 Participants

**Fig. 1:**
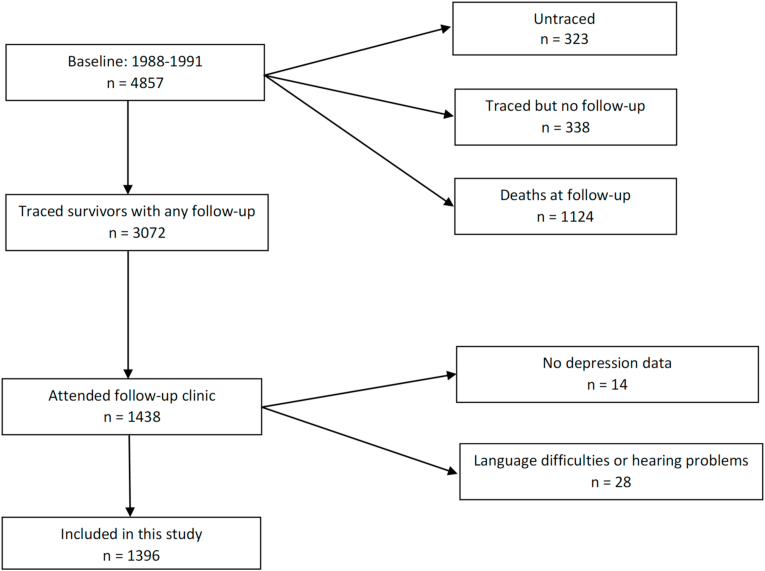
Flow chart showing sample size included in the 20 year follow-up study.

Participants were part of the ongoing tri-ethnic population-based longitudinal study, SABRE (Southall And Brent REvisited). Cohort members were recruited randomly from ethnicity-, sex- and age-stratified general practitioners' lists and local workplaces from North and West London at baseline (1988–1991) [23]. This cross-sectional study is based on 1438 survivors who attended the 20 year follow-up clinic (2008–2011). We excluded 42 participants who either did not complete the depression scale or who had language difficulties or hearing problems which were likely to have interfered with the assessment. Therefore, 1396 respondents were included in the present study (Fig. 1). All procedures involving human subjects were approved by St Mary's Hospital Research Ethics Committee (07/H0712/109) in accordance with the Declaration of Helsinki. Written informed consent was obtained from all subjects.

### 2.2 Assessment of depression

Depressive symptoms were recorded by interviewers using the 10-item Geriatric Depression Scale (GDS-10) [24]. Individuals were asked to respond to 10 items about how they had been feeling recently. Scores range from 0 to 10, with higher total scores indicating more severe depressive symptoms. A 3/4 cut-off provides the optimal balance between sensitivity and specificity for mild to moderate depression [25]. Participants with a GDS-10 ≥ 4 and/or use of antidepressant medications/hypnotics/anxiolytics were considered as having depression, and those with a GDS-10 < 4 were categorised as not having depression.

### 2.3 Assessment of cardio-metabolic status and subclinical CVD

Established diabetes mellitus was defined from primary care record review, self-report of doctor-diagnosed diabetes, and receipt of antidiabetic medications. Participants without known diabetes underwent an oral glucose tolerance test to identify unrecognized diabetes. Hypertension was identified from primary care medical record review or self-report of hypertension with use of antihypertensive medications. Participants were also asked to report doctor diagnosed CHD, stroke or heart failure. Fasting bloods were analysed for cholesterol and high-density lipoprotein. Fat percent was measured using bioelectrical impedance analysis (Tanita TBF‐410 MA body composition analyzer). Serum interleukin-6 (IL-6) was measured using a high sensitivity ELISA (R & D Systems, Abingdon, UK).

CAC was measured in Agatston units (AU) using a Philips 64 slice computerised tomography scanner and was categorised into four levels (0 AU, 1–100 AU, >100–400 AU, >400 AU) [26]. Far wall left common cIMT was assessed using a Philips iE33 ultrasound machine with an 11-3 MHz linear transducer. Carotid artery plaque was identified according to the Mannheim consensus [27]. Carotid-femoral pulse wave velocity (PWV) was used as a measure of arterial stiffness according to recent consensus guidelines [28]. Cerebral Magnetic Resonance Imaging (MRI) was performed based on the Cardiovascular Health Study protocol [29]. This included sagittal T1‐weighted and axial T1‐weighted, proton density, and T2‐weighted images of 5‐mm thickness with no gaps. The first third of the scans were performed on a General Electric Signa HDxt 1.5 T scanner and the rest on a General Electric Discovery MR750 3 T scanner (GE Healthcare, Waukesha, WI). The BaMoS algorithm [30] was used to segment WMH and a probabilistic lesion map was integrated to produce lesion volume measurements [31]. Brain infarcts of at least 3 mm were identified [32]. Albumin and creatinine measured from urine samples were used to calculate the albumin:creatinine ratio (ACR). Retinopathy was identified according to the NHS Diabetic Eye Screening classification [33] from digital retinal photography obtained using a mydriatic Zeiss FF450+ fundus camera (Oberkochen, Germany). Echocardiography was performed using a Philips iE33 ultrasound machine (Philips, Amsterdam) with a 5.0 to 1.0 phased array transducer (S5-1). Details of the measurement have been previously described [34]. Left atrial diameter was obtained using 2-dimensional echocardiography and indexed to height (LADI). Transmitral flow velocity during the early filling phase (E) was examined by pulsed wave Doppler. Tissue Doppler imaging (TDI) was performed for measurement of early diastole (e’), setting the sample volume at the lateral and septal mitral annulus. Averages of three consecutive cycles measured by Doppler were obtained. The ratio of transmitral E and TDI e’ velocities (E/e’) was calculated as an indicator of left ventricular (LV) filling pressure, and diastolic function was assessed on the basis of e’ and E/e’. Serum NT-proBNP and high-sensitive cardiac troponin T were measured using an Elecsys 2010 electrochemiluminescence analyzer (Roche Diagnostics, Burgess Hill, UK) calibrated using the manufacturer's reagents.

### 2.4 Assessment of sociodemographic and behavioural characteristics

Sociodemographic and behavioural information was taken from self-report, including age, sex, ethnicity, years of education, occupation, smoking status, alcohol consumption and physical activity. Occupation was classified as non-manual or manual. Smoking history was categorised into three groups (never, former, current). Alcohol intake was recorded as the number of units consumed per week. Physical activity was estimated from total weekly energy expended (MJ) in sport, walking and cycling, using energy expenditure estimates applied to self-reported activity [35].

### 2.5 Statistical analysis

Statistical analyses were performed using Stata version 14.2 (StataCorp LP, College Station, TX). Participant characteristics for continuous data were reported as mean ± SD or median [interquartile range] for skewed data. With respect to categorical variables, descriptive statistics were reported as frequency and percentage within each category.

The number of missing values for covariates ranged from 0 to 367 (26.3%). The variables with most missing values were pulse wave velocity (n = 367, 26.3%), due to equipment failure, and retinopathy (n = 246, 17.6%), due to contra-indications for pupillary dilation (not related to depression or CVD). Assuming that data were missing at random, missing data were imputed using multiple imputation by chained equations (MICE) on sex subsamples, as we hypothesised that there may be sex differences in cardiovascular structure and function and severity of depressive symptoms. Forty imputed datasets were created and pooled results stratified by sex for effect estimates were reported thereafter.

We conducted negative binomial regression due to the over-dispersed GDS-10 total score. All continuous subclinical CVD variables were standardised for comparability of regression coefficients (NT-proBNP and troponin were log transformed and then standardised). The 11 subclinical CVD variables (with pre-specified potential confounders – see below) were separately included in multivariable regression models as independent variables with the total depression score as the dependent variable. Regression coefficients and 95% confidence intervals (CIs) were reported. Regression coefficients are interpreted as the difference between the log of expected counts of depression score for one unit or one level change in the explanatory variable. All the analyses were conducted for men and women separately and sex by subclinical CVD variable interactions were tested. Ethnicity interactions were also tested but no marked interactions were found. Model 1 was unadjusted; model 2 included sociodemographic and behavioural characteristics (age, ethnicity, years of education, occupation, smoking, alcohol consumption and physical activity); and model 3 additionally adjusted for diabetes, hypertension, cholesterol/HDL ratio, fat percent and IL-6. The total WMH volume measure was adjusted for intracranial volume. These analyses were repeated after participants with doctor diagnosed CHD, stroke or heart failure were excluded. Sensitivity analyses were conducted using a complete case analysis, or with depression as a binary outcome.

## 3 Results

### 3.1 Participant characteristics

Mean age of the sample was 69.5 years (range: 58–86 years), and 76.2% were male (Table 1). Hypertension was frequent (66.8%), while 31% of participants had diabetes mellitus and 31% had diagnosed CHD, stroke or heart failure. The median (interquartile range) of GDS-10 total score was 1 [0, 2] for men and 1 [0, 3] for women. When participants with GDS-10 ≥ 4 and/or use of antidepressant medications/hypnotics/anxiolytics were considered as having depression, the prevalence of depression in women was higher than in men (19.9% *vs* 16.2%). Men had generally more adverse subclinical CVD compared to women, but women had poorer LV diastolic function (e’ 7.7 ± 2.1 *vs* 8.5 ± 2.5; E/e’ 9.9 ± 3.5 *vs* 9.3 ± 3.3) and higher NT-proBNP (106 [53, 186] *vs* 87 [47, 183]).

**Table 1:** Characteristics of the study population. Observed data (no imputations).

Mean (s.d.), *n* (%) or median [25th, 75th centiles]	Complete sample (n = 1396)	Men (n = 1064)	Women (n = 332)	Sample excluding people with CHD, stroke or heart failure (n = 961)	Men (n = 713)	Women (n = 248)
**Sociodemographic and cardiovascular characteristics**						
Age, yr	69.5 (6.1)	69.7 (6.1)	69.0 (6.1)	68.8 (6.0)	68.9 (5.9)	68.3 (6.1)
Ethnicity, *n* (%)						
European	680 (48.7)	527 (49.5)	153 (46.1)	502 (52.2)	379 (53.2)	123 (49.6)
South Asian	490 (35.1)	423 (39.8)	67 (20.2)	295 (30.7)	253 (35.5)	42 (16.9)
African Caribbean	226 (16.2)	114 (10.7)	112 (33.7)	164 (17.1)	81 (11.4)	83 (33.5)
Education, yr	11.8 (3.2)	11.9 (3.2)	11.2 (3.1)	11.8 (3.2)	12.0 (3.1)	11.4 (3.3)
Manual occupation, *n* (%)	831 (60.2)	673 (63.5)	158 (49.4)	549 (57.7)	439 (61.8)	110 (45.6)
Smoking, *n* (%)						
Never	778 (56.1)	552 (52.3)	226 (68.1)	542 (56.7)	372 (52.5)	170 (68.6)
Former	520 (37.5)	435 (41.2)	85 (25.6)	353 (36.9)	289 (40.8)	64 (25.8)
Current	89 (6.4)	68 (6.5)	21 (6.3)	61 (6.4)	47 (6.6)	14 (5.7)
Alcohol, units/week	2 [0, 8]	4 [0, 10]	0 [0, 2]	3 [0, 8]	4 [1, 2]]	0 [0, 2]
Physical activity, MJ/week	9.6 (4.4)	9.9 (4.6)	8.5 (3.7)	10.0 (4.4)	10.4 (4.6)	8.9 (3.6)
Coronary heart disease, *n* (%)	348 (24.9)	291 (27.4)	57 (17.2)	0	0	0
Stroke, *n* (%)	78 (5.6)	59 (5.6)	19 (5.7)	0	0	0
Heart failure, *n* (%)	132 (9.6)	100 (9.6)	32 (9.9)	0	0	0
Diabetes, *n* (%)	432 (31.0)	330 (31.0)	102 (30.7)	250 (26.0)	180 (25.3)	70 (28.2)
Hypertension, *n* (%)	932 (66.8)	720 (67.7)	212 (63.9)	547 (56.9)	409 (57.4)	138 (55.7)
Cholesterol/HDL ratio	3.6 (1.0)	3.6 (1.0)	3.4 (1.0)	3.7 (1.0)	3.7 (1.0)	3.5 (0.9)
Fat percent, %	29.1 (8.2)	26.3 (6.4)	38.0 (6.8)	29.2 (8.2)	26.2 (6.4)	37.7 (6.7)
IL-6, pg/mL	1.2 [0.7, 1.8]	1.2 [0.8, 1.9]	1.2 [0.7, 1.8]	1.1 [0.7, 1.8]	1.1 [0.7, 1.8]	1.1 [0.7, 1.7]
**Depressive symptoms measures**						
Total Score for the Geriatric Depression Scale (10 items, range 0–10)	1 [0, 2]	1 [0, 2]	1 [0, 3]	1 [0, 2]	1 [0, 2]	1 [0, 2.5]
GDS-10 ≥ 4 and/or use of antidepressant medication/hypnotics/anxiolytics, *n* (%)	238 (17.1)	172 (16.2)	66 (19.9)	132 (13.7)	89 (12.5)	43 (17.3)
**Cardiovascular structure and function**						
Coronary artery calcium (Agatston Units), *n* (%)						
0	290 (23.8)	137 (15.1)	153 (48.9)	254 (26.8)	123 (17.5)	131 (53.5)
1–100	378 (31.0)	293 (32.3)	85 (27.2)	315 (33.2)	241 (34.2)	74 (30.2)
>100–400	274 (22.5)	232 (25.6)	42 (13.4)	207 (21.8)	182 (25.9)	25 (10.2)
>400	277 (22.7)	244 (26.9)	33 (10.5)	173 (18.2)	158 (22.4)	15 (6.1)
Carotid intima-media thickness, mm	0.9 (0.2)	0.9 (0.2)	0.9 (0.2)	0.9 (0.2)	0.9 (0.2)	0.9 (0.2)
Carotid plaque, *n* (%)	209 (16.2)	163 (16.5)	46 (15.1)	118 (13.2)	91 (13.7)	27 (11.7)
Pulse wave velocity, m/s	11.5 (3.7)	11.6 (3.6)	11.1 (4.1)	11.2 (3.6)	11.2 (3.4)	11.0 (4.0)
Total white matter hyperintensities volume	4322.0 [2552.2, 8421.2]	4500.4 [2603.2, 8423.8]	3735.4 [2405.4, 8402.4]	4143.8 [2390.6, 7524.5]	4242.5 [2410.8, 7644.8]	3595.0 [2380.3, 6886.2]
Intracranial volume, L	1.3 (0.1)	1.4 (0.1)	1.2 (0.1)	1.3 (0.1)	1.4 (0.1)	1.2 (0.1)
Brain infarcts, *n* (%)	258 (20.3)	204 (20.8)	54 (18.5)	146 (16.5)	110 (16.4)	36 (16.6)
Albumin:creatinine ratio	0.4 [0.2, 0.9]	0.4 [0.2, 1.0]	0.4 [0.3, 0.8]	0.4 [0.2, 0.8]	0.4 [0.2, 0.8]	0.4 [0.3, 0.7]
Retinopathy, *n* (%)	381 (33.1)	301 (33.7)	80 (31.3)	249 (30.9)	189 (31.1)	60 (30.2)
LADI, cm/m	2.4 (0.3)	2.4 (0.3)	2.4 (0.3)	2.3 (0.3)	2.3 (0.3)	2.3 (0.3)
e’	8.3 (2.4)	8.5 (2.5)	7.7 (2.1)	8.4 (2.3)	8.6 (2.4)	7.8 (2.0)
E/e’	9.4 (3.4)	9.3 (3.3)	9.9 (3.5)	9.0 (2.9)	8.8 (3.0)	9.4 (2.6)
NT-proBNP, ng/mL	90 [48, 184]	87 [47, 183]	106 [53, 186]	75 [44, 136]	71 [41, 124]	87 [51, 152]
Troponin, ng/L	7.1 [4.6, 11.3]	7.9 [5.3, 12.7]	4.8 [2.1, 7.5]	6.4 [4.0, 9.7]	7.1 [4.9, 10.5]	4.5 [2.1, 6.6]

CHD, coronary heart disease; HDL, high-density lipoprotein; IL, interleukin; GDS, Geriatric Depression Scale; LADI, left atrial diameter indexed to height; e’, peak velocity during early diastole; mitral E, mitral flow velocity during the early filling phase; NT-proBNP, N terminal prohormone brain natriuretic peptide.

The subgroup without diagnosed CVD had a lower prevalence of depression than the complete sample (13.7% *vs* 17.1%), and was generally healthier in terms of subclinical CVD (Table 1).

### 3.2 Associations between clinical and subclinical CVD and total depression score

Most measures of CVD were adversely associated with depression scores in men (Table 2). These associations were attenuated when adjusted for sociodemographic and health behavioural factors (model 2), in particular physical activity. Generally, additional adjustment for other cardiovascular risk factors made no major further impact (model 3). The strongest association, remaining robust to multivariable adjustment, was that with carotid plaque (regression coefficient 0.22 [95% CI 0.03, 0.40 in model 3]. Associations were generally similar for women, again with attenuation when adjusted for sociodemographic factors and health behaviours, in particular occupation and physical activity. Strongest associations were with LADI, e’ and CAC. The association between ACR, diastolic function measures and depression differed by sex, when tested as an interaction (*p* < 0.1). For example, the regression coefficient for LADI was 0.07 [95% CI -0.01, 0.15] in men, and 0.23 [95% CI 0.11, 0.35] in women in model 3.

**Table 2:** Associations between subclinical macro and microvascular disease and depression score.

	Regression coefficient (95% CI)					
	Men (n = 1064)			Women (n = 332)		
	Model 1: unadjusted	Model 2: age, ethnicity, years of education, occupation, smoking, alcohol and physical activity	Model 3: model 2 + diabetes, hypertension, cholesterol/HDL ratio, fat percent and IL-6	Model 1: unadjusted	Model 2: age, ethnicity, years of education, occupation, smoking, alcohol and physical activity	Model 3: model 2 + diabetes, hypertension, cholesterol/HDL ratio, fat percent and IL-6
CAC (0 AU, 1–100 AU, >100–400 AU, >400 AU)	0.07 (−0.0002, 0.15)	0.05 (−0.03, 0.12)	0.04 (−0.04, 0.11)	0.12 (0.02, 0.23)	0.12 (0.01, 0.23)	0.14 (0.03, 0.25)
cIMT (standardised)	0.02 (−0.06, 0.09)	−0.02 (−0.09, 0.05)	−0.01 (−0.08, 0.06)	0.10 (−0.02, 0.22)	0.06 (−0.06, 0.18)	0.07 (−0.06, 0.19)
Carotid plaque	0.21 (0.01, 0.40)	0.22 (0.03, 0.40)	0.22 (0.03, 0.40)	0.07 (−0.26, 0.40)	−0.08 (−0.41, 0.26)	−0.04 (−0.38, 0.31)
cfPWV (standardised)	0.04 (−0.04, 0.12)	−0.01 (−0.09, 0.07)	−0.01 (−0.09, 0.07)	−0.05 (−0.17, 0.08)	−0.07 (−0.21, 0.06)	−0.08 (−0.21, 0.06)
Total WMH volume (standardised)^a^	0.15 (0.08, 0.23)	0.08 (0.01, 0.16)	0.07 (−0.001, 0.15)	0.12 (0.01, 0.23)	0.09 (−0.03, 0.21)	0.11 (−0.01, 0.23)
Brain infarcts	0.26 (0.08, 0.44)	0.17 (−0.003, 0.34)	0.14 (−0.03, 0.32)	0.06 (−0.24, 0.36)	−0.04 (−0.35, 0.26)	−0.03 (−0.33, 0.28)
ACR (log transformed, standardised)	0.14 (0.07, 0.21)	0.05 (0.03, 0.17)	0.05 (−0.02, 0.12)	0.01 (−0.10, 0.12)	−0.07 (−0.16, 0.07)	−0.07 (−0.18, 0.05)
Retinopathy	0.09 (−0.07, 0.26)	0.03 (−0.13, 0.18)	0.01 (−0.15, 0.17)	0.21 (−0.06, 0.49)	0.13 (−0.15, 0.40)	0.14 (−0.13, 0.42)
LADI (standardised)	0.08 (0.005, 0.15)	0.05 (−0.02, 0.12)	0.07 (−0.01, 0.15)	0.24 (0.12, 0.35)	0.21 (0.10, 0.32)	0.23 (0.11, 0.35)
e’ (standardised)	−0.09 (−0.17, −0.02)	−0.03 (−0.11, 0.04)	−0.04 (−0.12, 0.03)	0.06 (−0.05, 0.17)	0.15 (0.04, 0.26)	0.16 (0.05, 0.27)
E/e’ (standardised)	0.12 (0.04, 0.20)	0.04 (−0.03, 0.12)	0.04 (−0.04, 0.11)	−0.02 (−0.14, 0.10)	−0.08 (−0.20, 0.04)	−0.07 (−0.19, 0.05)
NT-proBNP (log transformed, standardised)	0.10 (0.03, 0.17)	0.06 (−0.02, 0.13)	0.04 (−0.04, 0.12)	0.05 (−0.07, 0.17)	0.05 (−0.07, 0.17)	0.08 (−0.05, 0.21)
Troponin (log transformed, standardised)	0.20 (0.13, 0.27)	0.12 (0.04, 0.20)	0.12 (0.04, 0.20)	0.10 (−0.02, 0.22)	0.04 (−0.10, 0.17)	0.05 (−0.09, 0.18)

HDL, high-density lipoprotein; IL, interleukin; CAC, coronary artery calcium; AU, Agatston Units; cIMT, carotid intima-media thickness; cfPWV, carotid to femoral pulse wave velocity; WMH, white matter hyperintensities; ACR, albumin:creatinine ratio; LADI, left atrial diameter indexed to height; e’, peak velocity during early diastole; mitral E, mitral flow velocity during the early filling phase; NT-proBNP, N terminal prohormone brain natriuretic peptide.

a Additionally adjusted for intracranial volume in all models.

### 3.3 Associations between subclinical CVD and total depression score in participants without diagnosed CVD

In the sample restricted to those without diagnosed CVD, associations between macro and microvascular subclinical disease and depression scores remained, although sometimes weaker than that observed in the whole sample and with wide confidence limits (Table 3). Associations with microvascular disease were preserved to a greater extent than macrovascular disease.

**Table 3:** Subgroup without diagnosed CVD: associations between subclinical macro and microvascular disease and depression score.

	Regression coefficient (95% CI)					
	Men (n = 713)			Women (n = 248)		
	Model 1: unadjusted	Model 2: age, ethnicity, years of education, occupation, smoking, alcohol and physical activity	Model 3: model 2 + diabetes, hypertension, cholesterol/HDL ratio, fat percent and IL-6	Model 1: unadjusted	Model 2: age, ethnicity, years of education, occupation, smoking, alcohol and physical activity	Model 3: model 2 + diabetes, hypertension, cholesterol/HDL ratio, fat percent and IL-6
CAC (0 AU, 1–100 AU, >100–400 AU, >400 AU)	0.04 (−0.05, 0.13)	0.02 (−0.07, 0.12)	0.03 (−0.07, 0.13)	0.05 (−0.10, 0.20)	0.03 (−0.12, 0.19)	0.05 (−0.11, 0.21)
cIMT (standardised)	−0.07 (−0.18, 0.04)	−0.09 (−0.19, 0.02)	−0.07 (−0.17, 0.04)	0.11 (−0.05, 0.27)	0.04 (−0.12, 0.21)	0.03 (−0.14, 0.20)
Carotid plaque	0.22 (−0.05, 0.48)	0.28 (0.02, 0.53)	0.29 (0.03, 0.55)	0.07 (−0.36, 0.50)	−0.15 (−0.60, 0.29)	−0.08 (−0.53, 0.38)
cfPWV (standardised)	0.04 (−0.08, 0.15)	−0.02 (−0.13, 0.08)	−0.02 (−0.13, 0.09)	−0.08 (−0.25, 0.09)	−0.11 (−0.29, 0.06)	−0.11 (−0.29, 0.07)
Total WMH volume (standardised)^a^	0.10 (−0.01, 0.22)	0.08 (−0.04, 0.19)	0.07 (−0.05, 0.18)	0.05 (−0.13, 0.22)	0.03 (−0.15, 0.22)	0.05 (−0.14, 0.24)
Brain infarcts	0.12 (−0.13, 0.37)	0.13 (−0.11, 0.37)	0.12 (−0.12, 0.37)	−0.01 (−0.38, 0.37)	−0.08 (−0.45, 0.28)	−0.09 (−0.46, 0.28)
ACR (log transformed, standardised)	0.12 (0.02, 0.22)	0.05 (−0.05, 0.15)	0.06 (−0.05, 0.16)	−0.02 (−0.17, 0.13)	−0.10 (−0.25, 0.05)	−0.07 (−0.23, 0.08)
Retinopathy	−0.04 (−0.27, 0.19)	−0.09 (−0.30, 0.12)	−0.11 (−0.32, 0.11)	0.17 (−0.16, 0.51)	0.08 (−0.24, 0.41)	0.11 (−0.21, 0.44)
LADI (standardised)	0.02 (−0.09, 0.13)	0.01 (−0.09, 0.12)	0.05 (−0.07, 0.16)	0.20 (0.05, 0.35)	0.15 (0.005, 0.30)	0.21 (0.06, 0.37)
e’ (standardised)	−0.03 (−0.13, 0.07)	0.002 (−0.10, 0.10)	−0.01 (−0.11, 0.09)	0.05 (−0.09, 0.18)	0.16 (0.02, 0.30)	0.16 (0.02, 0.30)
E/e’ (standardised)	0.03 (−0.08, 0.15)	−0.04 (−0.15, 0.07)	−0.04 (−0.15, 0.08)	−0.05 (−0.24, 0.14)	−0.16 (−0.35, 0.03)	−0.15 (−0.34, 0.04)
NT-proBNP (log transformed, standardised)	0.01 (−0.11, 0.12)	−0.01 (−0.13, 0.11)	−0.02 (−0.14, 0.10)	−0.03 (−0.19, 0.14)	−0.02 (−0.19, 0.15)	0.01 (−0.17, 0.18)
Troponin (log transformed, standardised)	0.14 (0.04, 0.24)	0.09 (−0.02, 0.19)	0.10 (−0.004, 0.21)	0.11 (−0.05, 0.26)	0.04 (−0.13, 0.20)	0.05 (−0.12, 0.21)

HDL, high-density lipoprotein; IL, interleukin; CAC, coronary artery calcium; AU, Agatston Units; cIMT, carotid intima-media thickness; cfPWV, carotid to femoral pulse wave velocity; WMH, white matter hyperintensities; ACR, albumin:creatinine ratio; LADI, left atrial diameter indexed to height; e’, peak velocity during early diastole; mitral E, mitral flow velocity during the early filling phase; NT-proBNP, N terminal prohormone brain natriuretic peptide.

a Additionally adjusted for intracranial volume in all models.

### 3.4 Sensitivity analyses

After excluding participants with any missing values on covariates, the sample size greatly decreased to 396. Sensitivity analyses results based on complete case analysis were largely consistent with the primary analyses (Supplementary Table 1). Sensitivity analyses using GDS-10 score of 4 or higher and/or use of antidepressant medications/hypnotics/anxiolytics as a threshold for elevated depressive symptoms yielded similar findings (Supplementary Table 2).

## 4 Discussion

In a community-based, multi-ethnic sample, with and without established CVD, we found that most measures of macro and microvascular disease were associated with increased levels of depressive symptoms in both men and women. Sociodemographic and health behavioural factors, in particular physical activity, accounted for some of these associations. This result is in agreement with previous studies which found that physical inactivity is bi-directionally associated with depression [36] and CVD [37,38]. We interpret this as indicating that physical activity may be on the causal pathway between CVD and depression and may therefore partially mediate the association. Measured cardiovascular risk factors did not further attenuate our observed associations. The residual associations may suggest that macro and microvascular dysfunction represents an independent pathway in the development of depression, or vice versa. Our results support the Data Linkage Study which found associations between depression and twelve clinically diagnosed cardiac, cerebrovascular, and peripheral diseases [39], and imply a potential common pathophysiology. Understanding underlying mechanisms is essential as patients with both depression and CVD have poor depression outcomes, including persistence of depressive symptoms, unstable remission of depression, and high rate of resistance to antidepressants [40,41], as well as poor CVD outcomes [5]. In addition, we expand on previous results by showing that associations between most measures of subclinical CVD and depressive symptoms are also present in the sample restricted to those without diagnosed CVD, albeit sometimes weaker than in the complete sample. These findings suggest that CVD is linked to depression before cardiovascular symptoms develop. It is also notable that associations with microvascular disease were retained to a greater extent than macrovascular disease in the subgroup sample, probably because microvascular disease may precede macrovascular disease and promote atherosclerosis [42].

Although evidence for associations between subclinical macro and microvascular disease and depressive symptoms is convincing, specific underlying mechanisms are not well understood through a cross-sectional study. A recent meta-analysis of longitudinal studies found an association between white matter hyperintensities and incident depression [13], offering preliminary evidence to support the vascular depression hypothesis proposed by Alexopoulos [43]. The vascular depression hypothesis posits that a depressive syndrome in older adults can be predisposed, precipitated, or perpetuated by cerebrovascular disease [43]. However, no association was found between retinal microvascular diameters and incident major depressive disorder in a 9-year longitudinal study [44], and there is a lack of longitudinal data for other vascular subclinical disease. It is notable that depression may be caused by the cardiovascular event itself as a maladaptive response to stressful life events among participants with existing CVD [47]. Although the associations remained in those without diagnosed CVD, it is clearly known that macro and microvascular disease is associated with CVD. Therefore, we cannot rule out the possibility that people may be affected by perceived loss in terms of health, functional capacity, independence and so on if they are informed of established vascular disease [47]. Alternatively, participants may have been influenced by depression already before they suffered from vascular disease. As a matter of fact, depression might initiate or accelerate the progression of atherosclerosis through autonomic and hypothalamic-pituitary-adrenal axis dysregulation [48]. Apart from biological mechanisms, behavioural effect of depressive symptoms has been reported to be another potential mechanism for subclinical atherosclerosis, such as decreased physical activity, sleep disorders and smoking [49].

While the associations between measures of subclinical coronary disease, cerebrovascular disease, large vessel disease, myocardial injury and depressive symptoms were generally similar in men and women, our findings suggest that the associations for ACR were stronger in men and LADI were stronger in women. In the subgroup of participants without diagnosed CVD, we also noted sex differences consistent with those in the complete sample. No studies to date have investigated sex difference in the association between ACR and depression. The stronger association between LADI and depressive symptoms in women in our study might be linked to non-cardiac factors. One potential explanation may be anaemia. Women are more likely than men to be anaemic and low haemoglobin has been reported to be correlated with markers of diastolic dysfunction [50] and increased filling pressure [51]. Although haemoglobin was not measured at this clinic visit, measurement at a subsequent visit 5 years later indicated that SABRE study women, on average, had haemoglobin levels 1.2 mg/dl lower than men (13.3 vs 14.5 mg/dl) (unpublished data). It is possible that fatigue and lack of energy due to anaemia might masquerade as depressive symptoms, resulting in an ostensibly stronger relationship in women. Another potential mechanism for fatigue in women might be vitamin D deficiency and, although this was not measured in our study, it is frequently observed in older women [52].

This study builds on previous literature by examining a range of measures of CVD in relation to depression using extensive in depth phenotyping. Our findings support multifactorial associations between CVD and depressive symptoms which were not explained by any individual cardiovascular risk factor. Our results from the subgroup of participants without known CVD yielded similar results to the complete sample and therefore the results are unlikely to be driven by disability resulting from CVD or knowledge of diagnosis. Our study also expands previous work by exploring different potential associations in men and women.

Several limitations of our study deserve comment. First, our cross-sectional data make it impossible to examine the direction of underlying causal relationships. Covariates at one time point do not take into account cumulative or past exposure and we cannot exclude residual confounding. Second, multiple comparisons were made, increasing the possibility of a chance finding. Although the variables in our models were introduced based on a priori considerations, cautious interpretation is required given the multiple comparisons. Third, our sample may underrepresent people who were less healthy or socioeconomically disadvantaged. Fourth, our use of GDS-10 to measure depressive symptoms prevents us from contributing to the discussion of the relationship between subclinical CVD and different symptom dimensions of depression. Also, GDS-10 cannot distinguish between minor and major depression. Finally, although we have adjusted for some common diseases which are associated with depressive symptoms, we are unable to rule out all the individuals with other diseases or taking medications which might predispose them to depression.

In conclusion, our findings show that clinical and subclinical CVD are associated with depressive symptoms in a community–based sample of older men and women. These associations were not fully explained by established cardiovascular risk factors and appear to be present before clinically apparent CVD develop. Prospective longitudinal studies are needed to better understand the nature of the relationship between CVD and depression, particularly in women. Our findings suggest that CVD prevention is not only intrinsically important but if proven to be causal for depression, might offer additional benefits in terms of prevention of late-life depression. Conversely, intervention for depression also holds great promise in early prevention of CVD. In addition, the work reiterates importance of lifestyle factors, particularly physical activity, as an important upstream factor relevant to both CVD and depression. A healthy lifestyle including adequate physical activity may benefit people in preventing CVD and depression and provoking well-being. For patients with both CVD and depression, appropriate physical activity should be emphasized and active treatment for both diseases needs to be guaranteed in order to facilitate recovery.

## Financial support

The study was funded at baseline by the Medical Research Council, Diabetes UK, and 10.13039/501100000274British Heart Foundation and at follow-up by the 10.13039/100010269Wellcome Trust (082464/Z/07/Z) and 10.13039/501100000274British Heart Foundation (SP/07/001/23603, PG/08/103, PG/12/29/29497 and CS/13/1/30327). N.C. and A.D.H. receive support from the National Institute for Health Research University College London Hospitals Biomedical Research Centre.

## CRediT authorship contribution statement

**Jingyi Wang:** Conceptualization, Methodology, Software, Formal analysis, Writing - original draft, Visualization. **Therese Tillin:** Conceptualization, Methodology, Validation, Data curation, Writing - review & editing, Project administration. **Alun D. Hughes:** Conceptualization, Methodology, Writing - review & editing, Supervision, Funding acquisition. **Marcus Richards:** Conceptualization, Methodology, Writing - review & editing. **Naveed Sattar:** Conceptualization, Methodology, Writing - review & editing. **Chloe Park:** Conceptualization, Methodology, Writing - review & editing. **Nish Chaturvedi:** Conceptualization, Methodology, Writing - review & editing, Supervision, Funding acquisition.

## Declaration of competing interest

The authors declared they do not have anything to disclose regarding conflict of interest with respect to this manuscript.
