# Supplementary material for: Subclinical macro and microvascular disease is differently associated with depressive symptoms in men and women: Findings from the SABRE population-based study
Source: Atherosclerosis. 2020 Nov;312:35–42. doi: 10.1016/j.atherosclerosis.2020.09.005 (PMC7594642; doi:10.1016/j.atherosclerosis.2020.09.005)
Supplement: Multimedia component 1 [file mmc1.pdf]

# Supplementary material

**Supplementary Table 1** Associations between subclinical macro and microvascular disease and depression score. Complete case analysis

**Supplementary Table 2** Associations between subclinical macro and microvascular disease and depression (binary outcome). Pooled results after multiple imputation

This supplementary material has been provided by the authors to give readers additional information about their work.

**Supplementary Table 1.** Associations between subclinical macro and microvascular disease and depression score. Complete case analysis

| | Regression coefficient (95% CI) | | | | | |
|---|---|---|---|---|---|---|
| | **Men (n = 314)** | | | **Women (n = 82)** | | |
| | **Model 1: unadjusted** | **Model 2: age, ethnicity, years of education, occupation, smoking, alcohol and physical activity** | **Model 3: model 2 + diabetes, hypertension, cholesterol/HDL ratio, fat percent and IL-6** | **Model 1: unadjusted** | **Model 2: age, ethnicity, years of education, occupation, smoking, alcohol and physical activity** | **Model 3: model 2 + diabetes, hypertension, cholesterol/HDL ratio, fat percent and IL-6** |
| CAC (0 AU, 1-100 AU, >100-400 AU, >400 AU) | -0.03 (-0.17, 0.11) | -0.07 (-0.21, 0.07) | -0.11 (-0.26, 0.04) | 0.02 (-0.20, 0.24) | 0.01 (-0.21, 0.23) | 0.11 (-0.14, 0.36) |
| cIMT (standardised) | -0.09 (-0.23, 0.05) | -0.18 (-0.32, -0.03) | -0.16 (-0.31, -0.01) | 0.12 (-0.08, 0.33) | -0.05 (-0.25, 0.16) | 0.02 (-0.20, 0.23) |
| Carotid plaque | 0.23 (-0.14, 0.59) | 0.14 (-0.22, 0.50) | 0.11 (-0.26, 0.49) | 0.03 (-0.61, 0.66) | -0.21 (-0.81, 0.39) | -0.21 (-0.83, 0.40) |
| cfPWV (standardised) | 0.06 (-0.08, 0.20) | 0.005 (-0.14, 0.15) | -0.01 (-0.16, 0.14) | -0.04 (-0.26, 0.19) | -0.05 (-0.27, 0.18) | -0.01 (-0.25, 0.22) |
| Total WMH volume (standardised)[a] | 0.17 (0.04, 0.29) | 0.12 (-0.01, 0.25) | 0.10 (-0.03, 0.24) | 0.14 (-0.02, 0.31) | 0.12 (-0.06, 0.30) | 0.14 (-0.04, 0.32) |
| Brain infarcts | 0.32 (-0.03, 0.66) | 0.21 (-0.14, 0.56) | 0.12 (-0.24, 0.48) | 0.09 (-0.46, 0.63) | -0.02 (-0.51, 0.48) | 0.13 (-0.40, 0.66) |
| ACR (log transformed, standardised) | 0.09 (-0.04, 0.22) | 0.04 (-0.09, 0.18) | 0.03 (-0.11, 0.17) | 0.06 (-0.14, 0.27) | -0.02 (-0.22, 0.18) | -0.03 (-0.25, 0.19) |
| Retinopathy | -0.02 (-0.33, 0.28) | -0.01 (-0.31, 0.29) | -0.05 (-0.36, 0.26) | 0.77 (0.39, 1.15) | 0.57 (0.17, 0.96) | 0.59 (0.16, 1.01) |
| LADI (standardised) | 0.17 (0.01, 0.33) | 0.16 (0.001, 0.32) | 0.19 (0.02, 0.36) | 0.16 (-0.08, 0.40) | 0.06 (-0.15, 0.27) | 0.04 (-0.19, 0.28) |
| e’ (standardised) | -0.08 (-0.22, 0.07) | -0.02 (-0.18, 0.13) | -0.04 (-0.19, 0.12) | -0.06 (-0.25, 0.14) | 0.10 (-0.09, 0.30) | 0.07 (-0.15, 0.28) |
| E/e’ (standardised) | 0.12 (-0.05, 0.29) | 0.05 (-0.12, 0.23) | 0.02 (-0.15, 0.20) | 0.04 (-0.23, 0.31) | -0.17 (-0.44, 0.10) | -0.12 (-0.41, 0.17) |
| NT-proBNP (log transformed, standardised) | 0.10 (-0.04, 0.25) | 0.07 (-0.09, 0.23) | 0.03 (-0.13, 0.19) | -0.15 (-0.44, 0.13) | -0.06 (-0.31, 0.18) | -0.05 (-0.30, 0.21) |
| Troponin (log transformed, standardised) | 0.22 (0.07, 0.36) | 0.17 (0.01, 0.33) | 0.17 (0.01, 0.34) | 0.03 (-0.21, 0.26) | -0.11 (-0.36, 0.14) | -0.10 (-0.38, 0.18) |

HDL, high-density lipoprotein; IL, interleukin; CAC, coronary artery calcium; AU, Agatston Units; cIMT, carotid intima-media thickness; cfPWV, carotid to femoral pulse wave velocity; WMH, white matter hyperintensities; ACR, albumin:creatinine ratio; LADI, left atrial diameter indexed to height; e’, peak velocity during early diastole; Mitral E, mitral flow velocity during the early filling phase; NT-proBNP, N terminal prohormone brain natriuretic peptide.
a. Additionally adjusted for intracranial volume in all models.

**Supplementary Table 2.** Associations between subclinical macro and microvascular disease and depression (binary outcome). Pooled results after multiple imputation

| | **Relative risk (95% CI)[a]** | | | | | |
|---|---|---|---|---|---|---|
| | **Men (n = 1064)** | | | **Women (n = 332)** | | |
| | **Model 1: unadjusted** | **Model 2: age, ethnicity, years of education, occupation, smoking, alcohol and physical activity** | **Model 3: model 2 + diabetes, hypertension, cholesterol/HDL ratio, fat percent and IL-6** | **Model 1: unadjusted** | **Model 2: age, ethnicity, years of education, occupation, smoking, alcohol and physical activity** | **Model 3: model 2 + diabetes, hypertension, cholesterol/HDL ratio, fat percent and IL-6** |
| CAC (0 AU, 1-100 AU, >100-400 AU, >400 AU) | 1.08 (0.94, 1.24) | 1.02 (0.88, 1.19) | 1.04 (0.90, 1.22) | 1.20 (0.98, 1.47) | 1.20 (0.97, 1.48) | 1.23 (0.97, 1.54) |
| cIMT (standardised) | 1.00 (0.87, 1.15) | 0.97 (0.85, 1.11) | 1.00 (0.87, 1.15) | 1.16 (0.92, 1.47) | 1.09 (0.88, 1.36) | 1.11 (0.87, 1.41) |
| Carotid plaque | 1.27 (0.90, 1.80) | 1.31 (0.94, 1.83) | 1.34 (0.96, 1.88) | 0.78 (0.38, 1.62) | 0.64 (0.32, 1.28) | 0.68 (0.33, 1.37) |
| cfPWV (standardised) | 1.05 (0.90, 1.22) | 0.95 (0.81, 1.12) | 0.96 (0.82, 1.14) | 0.95 (0.76, 1.17) | 0.91 (0.71, 1.17) | 0.91 (0.71, 1.17) |
| Total WMH volume (standardised)[b] | 1.15 (1.02, 1.30) | 1.04 (0.90, 1.20) | 1.03 (0.89, 1.18) | 1.11 (0.89, 1.39) | 1.06 (0.86, 1.31) | 1.12 (0.90, 1.40) |
| Brain infarcts | 1.15 (0.82, 1.61) | 0.96 (0.68, 1.34) | 0.94 (0.67, 1.31) | 0.84 (0.44, 1.61) | 0.75 (0.38, 1.47) | 0.79 (0.40, 1.56) |
| ACR (log transformed, standardised) | 1.26 (1.12, 1.41) | 1.12 (1.00, 1.26) | 1.14 (1.02, 1.28) | 1.04 (0.84, 1.29) | 0.96 (0.77, 1.20) | 0.97 (0.78, 1.21) |
| Retinopathy | 1.23 (0.90, 1.69) | 1.12 (0.82, 1.53) | 1.14 (0.82, 1.59) | 1.28 (0.77, 2.14) | 1.24 (0.74, 2.08) | 1.31 (0.77, 2.22) |
| LADI (standardised) | 1.08 (0.94, 1.25) | 1.00 (0.88, 1.15) | 1.07 (0.94, 1.23) | 1.40 (1.16, 1.69) | 1.43 (1.16, 1.76) | 1.47 (1.17, 1.85) |
| e’ (standardised) | 0.87 (0.75, 1.02) | 0.92 (0.78, 1.09) | 0.89 (0.75, 1.05) | 1.17 (0.95, 1.44) | 1.28 (1.02, 1.59) | 1.30 (1.03, 1.63) |
| E/e’ (standardised) | 1.18 (1.05, 1.33) | 1.08 (0.95, 1.23) | 1.11 (0.98, 1.26) | 0.85 (0.66, 1.10) | 0.79 (0.63, 1.01) | 0.81 (0.63, 1.04) |
| NT-proBNP (log transformed, standardised) | 1.21 (1.06, 1.39) | 1.11 (0.97, 1.27) | 1.10 (0.96, 1.27) | 1.16 (0.92, 1.46) | 1.17 (0.91, 1.51) | 1.27 (0.96, 1.66) |
| Troponin (log transformed, standardised) | 1.29 (1.11, 1.50) | 1.14 (0.97, 1.34) | 1.17 (1.00, 1.37) | 1.09 (0.85, 1.40) | 1.07 (0.83, 1.39) | 1.10 (0.84, 1.43) |

HDL, high-density lipoprotein; IL, interleukin; CAC, coronary artery calcium; AU, Agatston Units; cIMT, carotid intima-media thickness; cfPWV, carotid to femoral pulse wave velocity; WMH, white matter hyperintensities; ACR, albumin:creatinine ratio; LADI, left atrial diameter indexed to height; e’, peak velocity during early diastole; Mitral E, mitral flow velocity during the early filling phase; NT-proBNP, N terminal prohormone brain natriuretic peptide.

a. Modified Poisson regression with a robust error variance was used to estimate relative risk.

b. Additionally adjusted for intracranial volume in all models.
